# Association between multiple-heavy-metal exposures and systemic immune inflammation in a middle-aged and elderly Chinese general population

**DOI:** 10.1186/s12889-024-18638-z

**Published:** 2024-04-29

**Authors:** Linhai Zhao, Yanfei Wei, Qiumei Liu, Jiansheng Cai, Xiaoting Mo, Xu Tang, Xuexiu Wang, Lidong Qin, Yujian Liang, Jiejing Cao, Chuwu Huang, Yufu Lu, Tiantian Zhang, Lei Luo, Jiahui Rong, Songju Wu, Wenjia Jin, Qinyi Guan, Kaisheng Teng, You Li, Jian Qin, Zhiyong Zhang

**Affiliations:** 1 Department of Occupational and Environmental Health, School of Public Health, Guangxi Medical University, Nanning, Guangxi Zhuang Autonomous Region China; 2 School of Public Health, Guilin Medical University, Guilin, Guangxi Zhuang Autonomous Region China; 3 Department of Epidemiology, School of Public Health (Guangdong Provincial Key Laboratory of Tropical Disease Research), Southern Medical University, Guangzhou, Guangdong China; 4 Guangxi Key Laboratory of Entire Lifecycle Health and Care, Guilin Medical University, Guilin, Guangxi Zhuang Autonomous Region China; 5 Guangxi Colleges and Universities Key Laboratory of Prevention and Control of Highly Prevalent Diseases, Guangxi Medical University, Nanning, Guangxi Zhuang Autonomous Region China; 6 Guangxi Key Laboratory of Environment and Health Research, Guangxi Medical University, Nanning, Guangxi Zhuang Autonomous Region China; 7 Key Laboratory of Longevity and Aging-related Diseases of Chinese Ministry of Education, Guangxi Medical University, Nanning, Guangxi Zhuang Autonomous Region China

**Keywords:** Heavy metals, Immunoinflammatory markers, Mixed exposure

## Abstract

**Background:**

Exposure to heavy metals alone or in combination can promote systemic inflammation. The aim of this study was to investigate potential associations between multiple plasma heavy metals and markers of systemic immune inflammation.

**Methods:**

Using a cross-sectional study, routine blood tests were performed on 3355 participants in Guangxi, China. Eight heavy metal elements in plasma were determined by inductively coupled plasma mass spectrometry. Immunoinflammatory markers were calculated based on peripheral blood WBC and its subtype counts. A generalised linear regression model was used to analyse the association of each metal with the immunoinflammatory markers, and the association of the metal mixtures with the immunoinflammatory markers was further assessed using weighted quantile sum (WQS) regression.

**Results:**

In the single-metal model, plasma metal Fe (log10) was significantly negatively correlated with the levels of immune-inflammatory markers SII, NLR and PLR, and plasma metal Cu (log10) was significantly positively correlated with the levels of immune-inflammatory markers SII and PLR. In addition, plasma metal Mn (log10 conversion) was positively correlated with the levels of immune inflammatory markers NLR and PLR. The above associations remained after multiple corrections. In the mixed-metal model, after WQS regression analysis, plasma metal Cu was found to have the greatest weight in the positive effects of metal mixtures on SII and PLR, while plasma metals Mn and Fe had the greatest weight in the positive effects of metal mixtures on NLR and LMR, respectively. In addition, blood Fe had the greatest weight in the negative effects of the metal mixtures for SII, PLR and NLR.

**Conclusion:**

Plasma metals Cu and Mn were positively correlated with immunoinflammatory markers SII, NLR and PLR. While plasma metal Fe was negatively correlated with immunoinflammatory markers SII, NLR, and PLR.

**Supplementary Information:**

The online version contains supplementary material available at 10.1186/s12889-024-18638-z.

## Introduction

In the complex immune response, inflammation is a protective response to a noxious stimulus that aims to remove the causative agent of injury, clear degenerated and necrotic cells, and promote reparative functions to keep the normal physiological activities of tissues and organs. When the inflammatory response persists, it can result in a variety of chronic diseases that can be harmful to human health [1]. White blood cell (WBC) counts and their subtypes are commonly used clinical indicators of systemic inflammation and are significantly elevated in most chronic inflammation-related diseases, such as diabetes and neoplasms [2, 3]. Most of the previous investigations have focused only on the relationship between individual cell types and inflammatory diseases. However, different blood cell types have different functions in chronic systemic inflammation. Therefore, a combination of inflammatory markers may better assess the association of chronic inflammation with the risk of disease development and death than a single inflammatory marker, as well as the interaction among inflammatory markers [4–7]. Five indicators of systemic immune inflammation based on the counting of leukocytes and their subtypes, including eosinophil–lymphocyte ratio (ELR), neutrophil–lymphocyte ratio (NLR), platelet–lymphocyte ratio (PLR), lymphocyte–monocyte ratio (LMR), and systemic immune-inflammation index (SII), are considered as relatively low-cost indicators of clinical inflammation that accurately reflect the status of systemic immune inflammation [8].

Exposure to heavy metals, including arsenic (As), cadmium (Cd), copper (Cu), iron (Fe), manganese (Mn), nickel (Ni), lead (Pb), and zinc (Zn), may affect the occurrence and development of inflammatory processes in the body. Potential routes of exposure to arsenic in the general population includes contamination of drinking water with industrial or agricultural chemical wastes or ingestion of preservatives containing inorganic arsenic [9]. Studies have shown that chronic arsenic exposure induces cardiovascular diseases through oxidative stress damage, inflammation, and endothelial dysfunction [10]. As for copper, zinc, and nickel, their main sources are drinking water and food. A retrospective study has shown that acute zinc deficiency causes a decrease in the immune system of the body, while chronic zinc deficiency increases the chances of an inflammatory reaction in the body [11]. In addition, excessive nickel exposure induces apoptosis and inhibits proliferation, thereby suppressing the development of immune organs [12], which reduces the number of T and B lymphocytes [13]. Moreover, occupational exposure to Pb was associated with significantly low LMR [14]. In a cross-sectional study, urinary cadmium levels were found to be negatively correlated with NLR and positively correlated with LMR [15]. Iron is an essential micronutrient for almost all living cells, and iron deficiency or excess is usually associated with inflammatory responses in the body [16]. Manganese is a trace essential metal necessary for the maintenance of human health and function, and the most common route of exposure to high levels of manganese is through inhalation of manganese from industrial sources. This may result in elevated levels of the metal in systemic tissues, which in turn disrupts the routine antioxidant activity of the MnSOD complex in mitochondria. This perturbation may affect the regulation of immune-inflammatory responses in humans [17, 18]. In addition, in a Chinese study, Zhong and his colleagues discovered that study participants with higher blood levels of manganese and cadmium had higher levels of SII [19]. Based on the abovementioned studies, we hypothesized that heavy metal exposure may have a potential association with immune-inflammatory markers in serum. To this point, findings on the relationship between indicators of immune inflammation (including SII, ELR, LMR, PLR, and NLR) and multiple-heavy-metal exposures are limited.

Therefore, we conducted a cross-sectional study among the general population of Gongcheng County, Guangxi, China. The area is rich in a variety of metals and reserves, such as iron, manganese, copper, and zinc, and mining plants are found in some areas, some of which have been shut down, but some of the mining methods used in the past have had an impact on the environment. Most of the county’s residents drink water from wells, so the potential for metal contamination is relatively high. Thus, the level of metal exposure for residents in the area is potentially different from that of other places. The group’s previous investigations found that plasma heavy metal levels of most of the residents in the area were associated with a variety of diseases [20–22], but the association with systemic immune inflammation remains unknown. Therefore, the present study was conducted to comprehensively evaluate the potential effects of heavy metal exposure on the body’s immune-inflammatory function by detecting the plasma concentrations of heavy metals (including As, Cd, Cu, Fe, Mn, Ni, Pb, and Zn) in the population and by measuring and calculating the levels of serum immune-inflammatory markers in the study population to standardize the use of these immune-inflammatory indicators in the population. The findings of this study have important implications for the full evaluation of the effects of multiple-heavy-metal exposure on systemic immune-inflammatory function.

## Materials and methods

### Study population

**Fig. 1:**
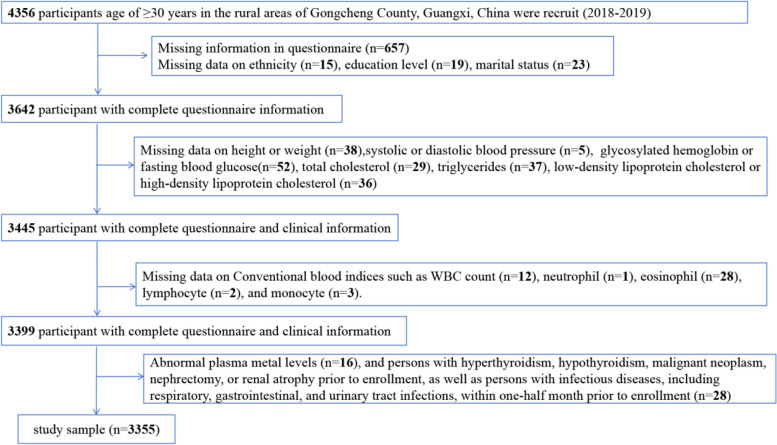
Flowchart of population included in our final analysis (*N* = 3355)

The study participants were from rural populations who voluntarily participated in the 2018–2019 health survey in Guilin Yao Autonomous County, Guangxi, southwestern China. The study participants were screened in accordance with the inclusion and exclusion standards. The inclusion standards were as follows: (1) voluntarily signed an informed consent form and (2) have lived in the study area for more than 5 years and are ≥ 30 years of age. The exclusion standards were as follows: (1) Persons with hyperthyroidism, hypothyroidism, malignant neoplasm, nephrectomy, or renal atrophy prior to enrollment, as well as persons with infectious diseases, including respiratory, gastrointestinal, and urinary tract infections, within one-half month prior to enrollment; (2) Persons with abnormal plasma metal levels (defined as a level of each metal greater than three times the 99th percentile); and (3) Persons who did not complete the questionnaire or incomplete information on the questionnaire. Finally, a total of 3355 study participants (1292 males and 2063 females) were included in the analysis (Fig. 1). The study has been approved by the Ethics Committee of Guilin Medical College (approval number: 20180702–3). Each of the participants provided a written informed consent.

### Assessment of plasma heavy metals

Plasma samples were stored in an ultra-low temperature refrigerator at − 80 °C until analysis. The concentrations of the eight plasma heavy metals selected for the study were measured using an inductively coupled plasma mass spectrometer (Thermo Fisher scientific iCAPRQ 01408, USA). Measurements have been described elsewhere [22]. Quality control of the instrument was performed in this study by measuring one quality control sample every 25 samples (ClinChek human plasma controls for trace metals Level 1 [No. 8883] and Level 2 [No. 8884]; Recipe Chemicals, Germany). Meanwhile, in this study, we controlled the recovery of metallic elements between 80 and 120%. When sample concentrations below the detection limit occurred, the detection limit/√2 was used instead [23]. The within-analytical coefficient of variation and among-analytical coefficient of variation of the eight plasma heavy metal concentrations selected for the study were found to be less than 10%. Therefore, they were included in the study.

### Definition of immune inflammation biomarkers

All study participants had their blood samples drawn from a vein early in the morning after 12 h of fasting, and then the blood samples were transported by cold-chain transport to the Laboratory Department of the County People’s Hospital for testing. Conventional blood indices such as WBC count, neutrophil, eosinophil, basophil, lymphocyte, monocyte, and platelet counts were measured using an automated clinical chemistry analyzer (Hitachi 7600–020, Kyoto, Japan) in accordance with the standardized criteria. The above indicators were set as parallel samples during testing to reduce errors. Immuno-inflammatory markers were calculated in accordance with the following formulas: NLR = neutrophil count (10^9^/L)/lymphocyte count (10^9^/L); PLR = platelet count (10^9^/L)/lymphocyte count (10^9^/L); LMR = lymphocyte count (10^9^/L)/monocyte count (10^9^/L); ELR = eosinophil count (10^9^/L)/lymphocyte count (10^9^/L); SII = neutrophil count (10^9^/L)/lymphocyte count (10^9^/L) × platelet count (10^9^/L) [24]. These markers reflect the overall inflammatory state of the individual body [8, 25].

### Definition of covariates

Participant’s socio-demographic characteristics and lifestyle habits were collected by trained interviewers through face-to-face interviews (Supplementary file), which included gender (male or female), age (30–59 years or ≥ 60 years), ethnicity (Yao or other), marital status (married or single/divorced/widowed), level of education (no education, 1–6 years or > 7 years and above), smoking (yes or no), and alcohol consumption (yes or no). Smoking is defined as smoking at least one cigarette a day for at least six months. Alcohol consumption is defined as drinking at least 50 g of alcohol or more per month for at least six months. The body mass index (BMI) was calculated on the basis of weight and height data: BMI = weight (kg)/height^2^ (m^2^). Underweight was defined as BMI < 18.5 kg/m^2^; normal weight was defined as 18.5 kg/m^2^ ≤ BMI ≤ 23.9 kg/m^2^; and overweight and obesity were defined as BMI ≥ 24.0 kg/m^2^ [26]. Diabetes was defined as one or more of the following conditions or taking glucose-lowering medication: glycosylated hemoglobin (HbA1c) ≥ 6.5% or fasting blood glucose ≥ 7.0 mmol/L [27]. Hypertension was defined as one or more of the following conditions or currently taking antihypertensive medication: systolic blood pressure ≥ 140 mmHg and/or diastolic blood pressure ≥ 90 mmHg [28]. Dyslipidemia was defined as one or more of the following conditions and/or taking lipid-lowering medications: total cholesterol ≥ 6.22 mmol/L, triglycerides ≥ 2.26 mmol/L, low-density lipoprotein cholesterol ≥ 4.14 mmol/L, and high-density lipoprotein cholesterol < 1.04 mmol/L [20].

### Statistical analysis

We described and analyzed all demographic and clinical characteristics of the study participants. In particular, normality test of the data was performed by using methods such as the Kolmogorov–Smirnov test and histograms. Continuous variables were described by mean ± standard deviation (mean ± SD) if normally distributed and median (25th percentile, 75th percentile) if not normally distributed. Comparisons between two groups were conducted by using t-test or Wilcoxon rank sum test. Categorical variables were described by frequency (n%), and comparisons between two groups were performed using the chi-square test or Fisher’s exact probability method. In addition, the heavy metal level data were skewed. Thus, the levels of all heavy metals were log_10_ (lg) transformed to bias reduction.

Then, Spearman’s correlation analysis was used to confirm the existence of a correlation among heavy metals. Generalized linear regression models were developed to assess the linear relationship between each heavy metal variable (log_10_-transformed continuous variables and quartiles) and immunoinflammatory biomarkers (raw continuous variables, including SII, NLR, PLR, LMR, and ELR). Models were adjusted for control variables such as sex, age, race, education, marital status, BMI, smoking, alcohol consumption, hypertension, dyslipidemia, and diabetes. False-positive rate controlled by Benjamini & Hochberg method. In addition, the estimation of the combined effect of multiple heavy metal exposures in plasma was performed by WQS regression, and significant individual components of mixtures contributing most to the effect on immunoinflammatory biomarkers were identified. In our study, we analyzed samples using two sets of WQS regression models designed to explore positive or negative correlations between metal mixtures and immunoinflammatory biomarkers. The parameters of each model were set as follows: 40% of the data were used as the test set and the remaining 60% as the validation set with 1000 bootstrap steps. In addition, to optimize the weight estimation, we applied positive or negative constraints in the optimization function. The WQS model was adjusted for gender, age, Ethnicity, Educated, BMI, Smoking, Drinking, Marital status, Diabates, Dyslipidemia, and Hypertension.

All *P*-values are two-sided with a statistically significant level of 0.05. All the above analyses were performed using IBM SPSS Statistics version 27 and R Studio version 4.2.1.

## Results

### Basic characteristics

A total of 3355 participants were included in this study, of which 61.5% were female and 38.5% were male. Table 1 shows the baseline characteristics of the study population stratified in accordance with gender. Among the baseline characteristics of the study population, a significant difference (*P* < 0.05) in all the characteristics of the study population was observed except for diabetes mellitus among the different genders. Among them, the male population generally had a higher level of education than the female population, and they were more likely to smoke and drink alcohol. However, the rates of hypertension and diabetes were higher in the female population than in the male population. The median levels of MONO, WBC, EOS, and NEU were higher in the male population than in the female population, whereas the median levels of PLT and LYM were higher in the female population than in the male population.

**Table 1:** Characteristics of the study participants stratified by genders (*n* = 3355)

Variable	Total	Male	Female	*P*-value^*^
	(*n* = 3355)	(*n* = 1292)	(*n* = 2063)	
Age [Years, n(%)]				< 0.001^b^
30–59	1672 (49.84)	567 (43.89)	1105 (53.56)	
≥ 60	1683 (50.16)	725 (56.11)	958 (46.44)	
Ethnicityn [n(%)]				0.008^b^
Yao	2506 (74.69)	998 (77.24)	1508 (73.10)	
Others	849 (25.31)	294 (22.76)	555 (26.90)	
Educated [n(%)]				< 0.001^b^
No formal educated	502 (14.96)	92 (7.12)	410 (19.87)	
1–6 years	1639 (48.85)	572 (44.27)	1067 (51.72)	
7 or more years	1214 (36.18)	628 (48.61)	586 (28.41)	
BMI [kg/m^2^, n(%)]				< 0.001^b^
< 18.5	267 (7.96)	66 (5.11)	201 (9.74)	
18.5–23.9	2035 (60.66)	808 (62.54)	1227 (59.48)	
≥ 24	1053 (31.39)	418 (32.35)	635 (30.78)	
Marital status [n(%)]				0.004^b^
Married	2817 (83.96)	1115 (86.30)	1702 (82.50)	
Single/divorced/widowed	538 (16.04)	177 (13.70)	361 (17.50)	
Smoking [n(%)]				< 0.001^b^
No	2723 (81.16)	668 (51.70)	2055 (99.61)	
Yes	632 (18.84)	624 (48.30)	8 (0.39)	
Alcohol consumption [n(%)]				< 0.001^b^
No	2285 (68.11)	596 (46.13)	1689 (81.87)	
Yes	1070 (31.89)	696 (53.87)	374 (18.13)	
Diabates [n(%)]				< 0.001^b^
No	2954 (88.05)	1095 (84.75)	1859 (90.11)	
Yes	401 (11.95)	197 (15.25)	204 (9.89)	
Dyslipidemia [n(%)]				0.873
No	944 (28.14)	361 (27.94)	583 (28.26)	
Yes	2411 (71.86)	931 (72.06)	1480 (71.74)	
Hypertension [n(%)]				0.034^†^
No	1825 (54.40)	733 (56.73)	1092 (52.93)	
Yes	1530 (45.60)	559 (43.27)	971 (47.07)	
PLT [10^9^/L, median [Q1; Q3]]	225.00 [191.00; 263.00]	209.00 [179.00; 245.00]	236.00 [201.00; 274.00]	< 0.001^a^
MONO [10^9^/L, median [Q1; Q3]]	0.39 [0.32; 0.49]	0.44 [0.35; 0.55]	0.37 [0.31; 0.46]	< 0.001^a^
WBC [10^9^/L, median [Q1; Q3]]	6.08 [5.15; 7.26]	6.14 [5.17; 7.53]	6.03 [5.12; 7.11]	0.001^a^
LYM [10^9^/L, median [Q1; Q3]]	1.78 [1.46; 2.17]	1.72 [1.38; 2.12]	1.81 [1.50; 2.19]	< 0.001^a^
EOS [10^9^/L, median [Q1; Q3]]	0.12 [0.07; 0.20]	0.14 [0.08; 0.24]	0.11 [0.07; 0.18]	< 0.001^a^
NEU [10^9^/L, median [Q1; Q3]]	4.04 [3.30; 5.00]	4.16 [3.39; 5.32]	3.98 [3.24; 4.86]	< 0.001^a^
SII [median [Q1; Q3]]	508.35 [373.65; 702.56]	513.24 [367.98; 712.60]	506.53 [376.20; 697.18]	0.644
NLR [median [Q1; Q3]]	2.27 [1.77; 2.97]	2.45 [1.89; 3.21]	2.19 [1.69; 2.83]	< 0.001^a^
PLR [median [Q1; Q3]]	126.47 [100.57; 158.63]	122.45 [95.23; 156.36]	128.71 [104.46; 160.12]	< 0.001^a^
LMR [median [Q1; Q3]]	4.54 [3.60; 5.69]	3.98 [3.11; 4.94]	4.97 [4.00; 6.04]	< 0.001^a^
ELR [median [Q1; Q3]]	0.07 [0.04; 0.11]	0.08 [0.05; 0.13]	0.06 [0.04; 0.10]	< 0.001^a^

*PLT* platelet count, *MONO* absolute monocyte count, *WBC* white blood cell count, *LYM* absolute lymphocyte count, *EOS* Absolute eosinophil count, *NEU* absolute neutrophil count, *ELR* eosinophil-lymphocyte ratio, *LMR* lymphocyte-monocyte ratio, *PLR* platelet-lymphocyte ratio, *NLR* neutrophil–lymphocyte ratio, *SII* Systemic Immune-Inflammation Index

^*^Statistical significance was set at *P* < 0.05 and marked in bold

^a^The Wilcoxon rank sum test was used to compare non-normally distributed data between groups

^b^The chi-square test was used to compare categorical variables

In addition, significant differences (*P* < 0.05) in the median level of the immunoinflammatory biomarkers NLR, PLR, LMR, and ELR were found between the two populations. Among them, the median levels of the immunoinflammatory biomarkers NLR and ELR were higher in the male population than in the female population, whereas the median levels of PLR and LMR were higher in the female population than in the male population.

### Plasma heavy metal concentrations and correlation

Table 2 demonstrates the plasma levels of heavy metals in different gender populations. The results showed significant differences in the plasma levels of metals Fe, Ni, Cu, As, and Pb between the two gender populations (all *P* < 0.05). Among them, the median plasma levels of metals Fe, Ni, As, and Pb were higher in the male population than in the female population, and the median plasma levels of metal Cu were higher in the female population than in the male population.

**Table 2:** Plasma levels of heavy metal elements in study populations by genders (*n* = 3355)

Plasma Heavy Metals [µg/L, median [Q1; Q3]]	Total	Male	Female	*P*-value^*^
	(*n* = 3355)	(*n* = 1292)	(*n* = 2063)	
Iron, mg/L	1.08 [0.83; 1.35]	1.29 [0.95; 1.50]	1.00 [0.76; 1.25]	< 0.001^a^
Nickel	5.08 [4.18; 6.16]	5.40 [4.40; 6.52]	4.89 [4.04; 5.87]	< 0.001^a^
Copper, mg/L	0.92 [0.80; 1.04]	0.87 [0.77; 0.99]	0.94 [0.84; 1.06]	< 0.001^a^
Zinc, mg/L	1.05 [0.76; 4.33]	1.06 [0.76; 4.37]	1.04 [0.76; 4.32]	0.458
Arsenic	1.19 [0.92; 1.95]	1.23 [0.94; 1.89]	1.17 [0.91; 2.00]	0.044^a^
Cadmium	0.19 [0.13; 0.27]	0.19 [0.13; 0.29]	0.19 [0.13; 0.27]	0.477
Lead	5.16 [3.32; 8.64]	5.74 [3.61; 9.18]	4.80 [3.17; 8.22]	< 0.001^a^
Manganese	2.12 [1.56; 2.97]	2.12 [1.57; 3.04]	2.11 [1.55; 2.93]	0.410

^*^Statistical significance was set at *P* < 0.05 and marked in bold

^a^The Wilcoxon rank sum test was used to compare non-normally distributed data between groups

**Fig. 2:**
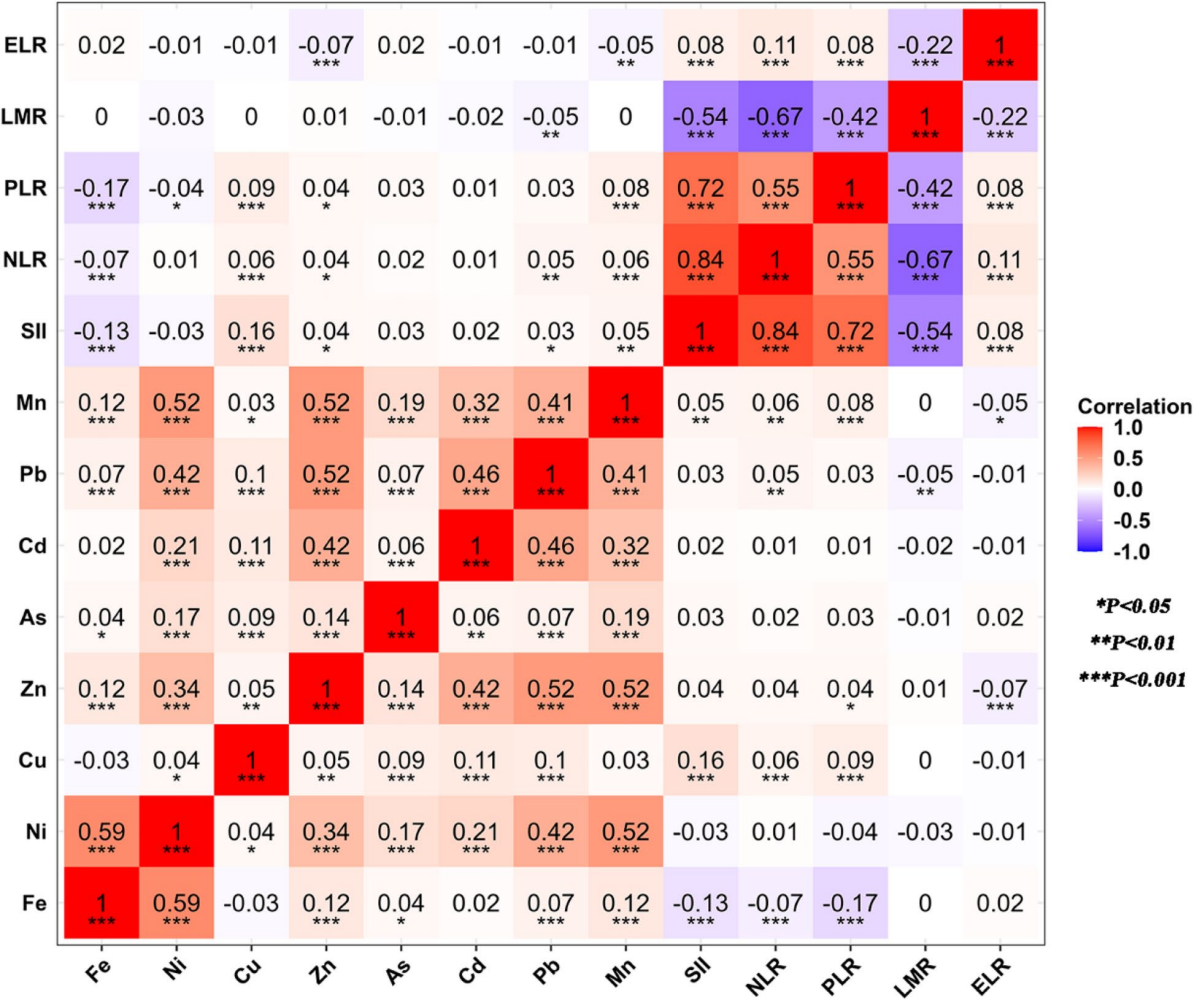
Heat map of association between plasma heavy metal concentrations (log_10_ conversion) and immune-inflammatory biomarkers in 3355 participants. ELR: eosinophil-lymphocyte ratio; LMR: lymphocyte-monocyte ratio; PLR: platelet-lymphocyte ratio; NLR: neutrophil–lymphocyte ratio; SII: Systemic Immune-Inflammation Index

The results of the correlations between the eight heavy metals and the immunoinflammatory biomarkers are shown in Fig. 2, where the correlations between the metals and the immunoinflammatory biomarkers ranged from negative to positive (− 0.17 to 0.16). Among them, a positive correlation was found between Cu and SII (*r* = 0.16), and a negative correlation was found between Fe and PLR (*r* =  − 0.17).

### Association between immune inflammation biomarkers and plasma heavy metals

#### Single effect of heavy metal exposure on immune inflammation biomarkers

**Fig. 3:**
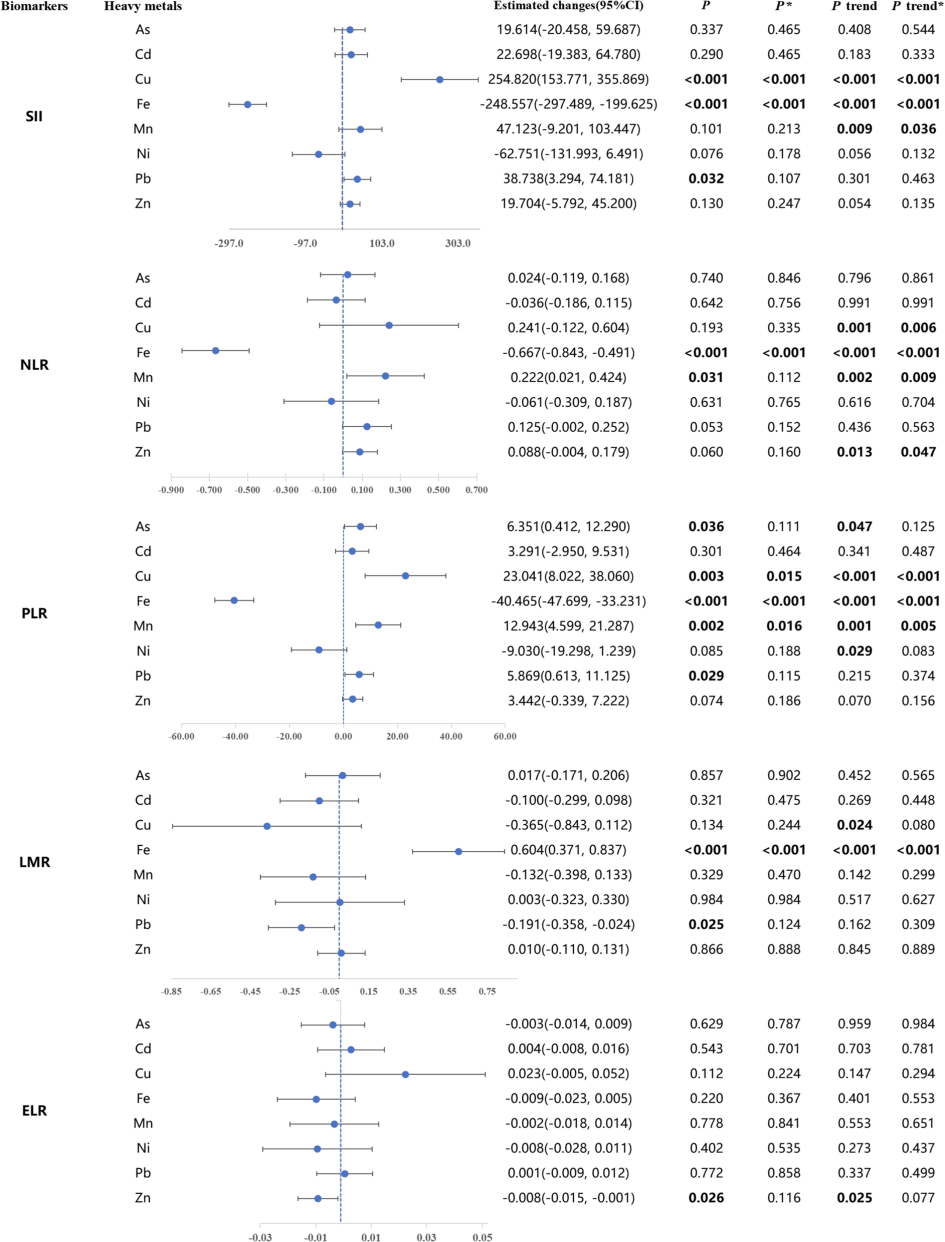
Generalized linear regression model-based investigation of the effect of single heavy metal exposures (all log10 transformed) on immunoinflammatory biomarkers. All above are adjusted for gender, age, Ethnicity, Educated, BMI, Smoking, Alcohol consumption, Marital status, Diabates, Dyslipidemia, and Hypertension. *β* is the change in the standardized systemic inflammatory index per 1 SD increase in log_10_-converted plasma heavy metal concentrations. The value of *β* at the blue dashed line is 0. *P* trend was examined by using the median of each quartile of plasma heavy metals as a continuous variable in the model. ^*^P value/*P* trend in multiple testing. ELR: eosinophil-lymphocyte ratio; LMR: lymphocyte-monocyte ratio; PLR: platelet-lymphocyte ratio; NLR: neutrophil–lymphocyte ratio; SII: Systemic Immune-Inflammation Index

As shown in Fig. 3, plasma metal Fe was discovered to be significantly negatively correlated with the levels of immune inflammatory markers SII, NLR and PLR. In the monometallic model, after adjusting for potential confounders, when plasma heavy metals were used as a continuous variable, for every 1 SD unit increase in the log_10_-transformed level of plasma Fe, the levels of immunoinflammatory markers SII, NLR, and PLR (95% CI) were decreased to − 248.557 (− 297.489, − 199.625), − 0.667 (− 0.843, − 0.491), − 40.465 (− 47.699, − 33.231). Meanwhile, the levels of immuno-inflammatory markers SII, NLR and PLR were significantly decreased with increasing quartiles of plasma metal Fe (log_10_ conversion) after multiple corrections (all *P* trend < 0.001). Plasma metal Cu was significantly and positively correlated with the levels of immune inflammatory markers SII and PLR. In the monometallic model, after adjusting for potential confounders, each 1 SD unit increase in the log_10_-transformed level of plasma Cu was associated with an increase in the levels (95% CI) of the immunoinflammatory markers SII and PLR of 254.820 (153.771, 355.869), 23.041 (8.022, 38.060). Meanwhile, the levels of immuno-inflammatory markers SII and PLR were significantly decreased with increasing quartiles of plasma metal Cu (log_10_ converted) after multiple corrections (all *P* trend < 0.001). Plasma metal Mn (log_10_-converted) as a continuous variable was significantly and positively correlated with the levels of immune inflammatory markers NLR and PLR. After multiple corrections, the levels of the immune inflammatory markers NLR (*P* trend = 0.009) and PLR (*P* trend = 0.005) were significantly increased with increasing quartiles of plasma metal Mn (log_10_ conversion).

In addition, plasma metal Pb (log_10_ conversion) as a continuous variable was significantly positively correlated with the levels of immune inflammatory markers SII and PLR, and significantly negatively correlated with the levels of LMR, but this association disappeared after multiple corrections. Similarly, plasma metal Zn (log_10_-converted) as a continuous variable was significantly negatively correlated with levels of the immunoinflammatory marker ELR, but this association disappeared after multiple corrections.

#### Combined effects of heavy metal mixture exposure on immune inflammation biomarkers

**Fig. 4:**
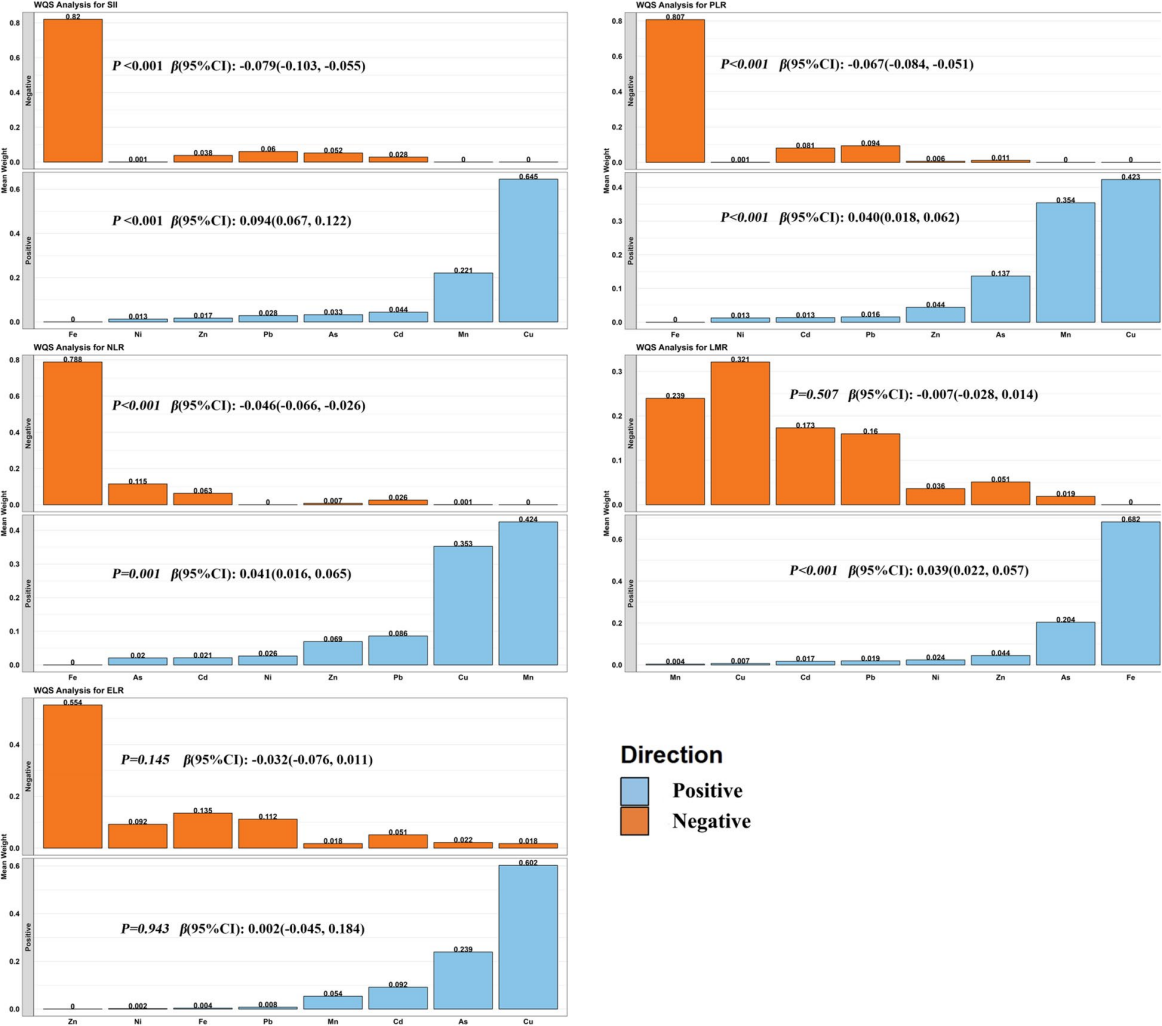
Positive or negative associations between heavy metal mixture exposure (all log transformed) and immunoinflammatory biomarkers were explored based on WQS regression modeling. The model was adjusted for gender, age, Ethnicity, Educated, BMI, Smoking, Alcohol consumption, Marital status, Diabates, Dyslipidemia, and Hypertension. ELR: eosinophil-lymphocyte ratio; LMR: lymphocyte-monocyte ratio; PLR: platelet-lymphocyte ratio; NLR: neutrophil–lymphocyte ratio; SII: Systemic Immune-Inflammation Index

We used a WQS regression model to explore the combined effects of metal mixtures on immunoinflammatory biomarkers (Fig. 4). The results showed that the mixture exposure of total metals significantly increased SII [*β*(95%CI): 0.094 (0.067, 0.122)], NLR [*β*(95%CI): 0.041 (0.016, 0.065)], PLR [*β*(95%CI): 0.040 (0.018, 0.062)], and LMR in the positive WQS model [*β* (95%CI): 0.039 (0.022, 0.057)] levels. Among them, plasma Cu had the greatest weight in the positive effects of mixed metals on SII (0.645) and PLR (0.423), and plasma Fe (0.682) had the greatest weight in the positive effects of mixed metals on LMR. In addition, plasma Mn (0.424) had the greatest weight in the positive effect of mixed metals on NLR, while Cu had a weight of 0.353.

Meanwhile, mixed exposure to total metals significantly increased the negative WQS model SII [*β*(95% CI): − 0.079 (− 0.103, − 0.055], PLR [*β*(95% CI): − 0.067 (− 0.084, − 0.051)], NLR [*β*(95% CI): − 0.046 (− 0.066, − 0.026)] levels. Among them, plasma Fe had the greatest weight in the negative effect of mixed metals on SII (0.820), PLR (0.807), and NLR (0.788).

## Discussion

The inflammatory response is a self-protective mechanism produced by the organism in response to external stimuli or infections. However, chronic inflammatory responses can lead to a combination of organizational damage, pain, changes in pathology, and the development of a pathological microenvironment [29]. In accurately evaluating the severity of inflammation, the researchers have developed a series of inflammatory markers [2, 8, 30]. As a biomarker of inflammation and immune response, peripheral WBC count and its subtypes are closely related to the development of many diseases. However, past studies have focused on the correlation between different subtype counts and diseases. In addition, different types of blood cells play different roles in the systemic inflammatory response. Moreover, based on the results of previous studies conducted by the group, a correlation between the presence of heavy metals and a variety of diseases was found in our study participants [20–22], which are closely related to inflammation [31, 32]. Meanwhile, metal exposure induces an inflammatory response in the organism, which is manifested by different degrees of changes in blood cell levels [33, 34]. However, changes in these conventional indicators often do not provide enough information to fully explain the complex mechanisms of inflammatory responses induced by metal exposure. In order to more comprehensively assess the effects of metal exposure on the body's immune system, a set of comprehensive indicators based on the counting of leukocytes and their subtypes were used in this study. These comprehensive indices take into account the changes in different cell types, and thus can more accurately reflect the overall effect of the inflammatory response induced by metal exposure. Compared with single indicators, this set of composite indicators has higher sensitivity and specificity in assessing the association between metal exposure and immune function. It can help us better understand the regulation of the immune system by metal exposure and reveal possible interactions and mechanisms of influence.

Iron is an essential nutrient, but in excess it can have deleterious effects. Excess iron can contribute to an increase in free iron, resulting in increased oxidative stress, as well as the production of a range of free radicals and reactive oxygen species that damage cellular structure and function [35, 36]. This oxidative stress may be damaging to cells, tissues and organs and result in increased inflammatory responses [37]. Meanwhile, iron is the fundamental element for neutrophil functioning [38], and the iron-dependent metalloprotein myeloperoxidase in neutrophils exerts antimicrobial effects through its Fe^3+^/Fe^2+^ redox state [39]. In addition, recent studies have shown that iron inhibits Th1 cell differentiation and interferon-γ (IFN-g) expression [40]. Thus, there is a correlation between iron and inflammation. However, previous studies have focused only on the association between iron levels and single inflammatory indicators (e.g., single blood cell levels), and a single indicator represents the level of immune inflammation in vivo. In contrast, our study focused on more than just the effect of iron on single inflammatory blood cell levels. The results of our study showed a significant negative correlation association between plasma metallic iron levels and inflammatory markers such as SII, NLR, and PLR. Consistent with our study, Yang et al. showed that higher or lower SII may be associated with recognition of iron overload and iron deficiency [41]. A negative correlation between plasma levels of metallic iron and immunoinflammatory markers, such as SII, NLR, MLR, and PLR, was also found in Xu et al.’s study [42]. In a study conducted by Zhou et al., a significantly negative correlation was found between plasma iron levels and NLR [43]. Therefore, high iron levels may inhibit the elevated levels of immune inflammation in vivo.

Copper (Cu) is a major trace element, and the homeostatic regulatory mechanism of Cu levels in the body is important for the maintenance of cellular homeostasis in inflammatory states. The disruption of Cu homeostasis in the body may trigger an adverse inflammatory response [44, 45]. Studies have shown that zinc finger protein 36 (Tristetraprolin, TTP) plays an important role in regulating the inflammatory response, and when Cu concentration increases, some of the Cu is induced to bind to TTP, which prevents TTP from inhibiting the inflammatory response, resulting in increased inflammation levels [46]. Animal experiments have shown that the addition of metals such as Cu to the diet enhances the immunity of dairy cows in the pre-and post-partum period and increases the percentage of neutrophils by promoting neutrophil–endothelial interactions, which reduces the mobility of polymorphonuclear neutrophils and causes them to adhere to the endothelium [47, 48]. The above studies have also demonstrated that changes in copper levels in vivo can significantly affect changes in the levels of single inflammatory cells. The five composite indices chosen for our study, which are based on the calculation of WBC and their subtype counts, have elevated levels that are essentially a combined increase or decrease in WBC and their subtype counts. A pilot study showed a positive correlation between blood copper concentration and preoperative SII in coronary artery bypass grafting [49]. This finding is consistent with our findings. That is, Cu-induced oxidative stress may determine the inflammatory response, and elevated Cu levels may affect inflammatory mechanisms [50]. Nevertheless, there is some heterogeneity in the results of different studies, and a study including 93 participants showed that the association between plasma Cu levels and inflammatory markers of immunity was not significant [42], which may result from the small sample size of the study.

Manganese is an essential human metal and is critical for the regulation of protein synthesis, metabolism, neurotransmitter production and immune function [51, 52]. However, excessive manganese exposure is toxic to humans and contributes to the development of various health conditions [52]. Manganese exposure in the general population is mainly related to daily nutritional and water intake [53] and environmental exposures [54]. It has been shown that manganese exposure is positively associated with osteoporosis, and that the inflammatory response acts as a mediator in this association [55]. Furthermore, recent studies have identified that manganese exposure and related diseases affect elevated levels of oxidative stress and inflammation in vivo [56]. The present study was conducted to investigate the effect of heavy metal exposure alone and in combination on the collective immune-inflammatory response by using five composite metrics calculated based on counts of leukocytes and their subtypes to represent in vivo immune-inflammatory levels. The results showed that as plasma metal Mn (log_10_-converted) quartiles increased, so did the levels of the immune inflammatory markers SII, PLR and NLR, and this association persisted after multiple corrections. However, the study conducted by Xu et al. did not find a significant correlation between plasma manganese metal and SII, MLR, NLR, and PLR [42]. This contradictory result may be due to different populations, sample sizes (our study: *n* = 3355; Xu et al.’s study: *n* = 93), and plasma manganese doses (median plasma manganese metal level in our study: 2.12 μg/L; median plasma manganese metal level in Xu et al.’s study: 1.98 μg/L). Similarly, the study conducted by Zhou et al. found no significant correlation between plasma Mn metal and NLR [43]. This contradictory conclusion needs to be confirmed by further studies.

Zinc has been shown to play a key role in the regulation of inflammatory responses [57–59]. Nevertheless, the effect of zinc deficiency on the immune response system remains unknown with regard to changes in the distribution of total WBCs. Japanese researchers discovered that zinc deficiency increased the total number of leukocytes, granulocytes, and monocytes, but it did not alter the number of lymphocytes, T-lymphocytes, B-lymphocytes, or NK cells within 2–4 weeks [60]. Animal experiments have also demonstrated that zinc deficiency significantly influences the counts of total leukocytes, neutrophils, and eosinophils in rats, but their recovery response is reversible, and inflammatory and stress reactions may play an important role in their changes [61]. In our study, ELR its level was reduced essentially by a decrease in eosinophil count and an increase in lymphocyte count. However, in our study, we did not discover an association between zinc and any of the immunoinflammatory markers. Exposure to arsenic, cadmium, nickel, and lead affects the levels of inflammatory markers in the human organism in occupational populations and in populations living in areas contaminated with heavy metals [62–64]. However, evidence in the general population is limited, and the results of a cross-sectional study in China showed a significant positive association between plasma cadmium metal and SII, but did not discover an association between lead and SII. The study conducted by Yu et al. using a large population showed that early occupational lead exposure increased NLR and induced genotoxicity [65]. Exposure levels of arsenic, cadmium, and lead were higher among participants in these studies than in the current study.

To this point, our study has the following strengths. First, our study used a comprehensive index based on the counts of leukocytes and their subtypes to assess the inflammatory response induced by metal exposure. Compared with the traditional single index, this composite index more comprehensively considers changes in different cell types and accurately reflects the effects of metal exposure on immune function. Second, while several older investigations have examined the association between five systemic immune-inflammatory markers and exposure to heavy metals, our study can provide further insight into the relationship. Also, the large sample size supports a substantial association between immune inflammatory markers and heavy metal exposure alone and in combination. In addition, this study used extensive covariate data to account for potential confounding variables that may alter the relationship between blood metal element concentrations and immune inflammatory markers. However, this study has some limitations. First, ours was a cross-sectional study and therefore could not show causal or temporal relationships. Second, the baseline counts of leukocytes and their subtypes were one-time measurements and not representative of a long-term chronic systemic inflammatory state, which may lead to an underestimation of immune inflammatory markers associated with plasma metals. Finally, as the present study analysed a total of 40 combinations of exposures and outcomes by analysing associations between five immune inflammatory markers and eight plasma heavy metals. We therefore used the Benjamini & Hochberg method to control for false discovery rates. However, this resulted in the loss of many statistically significant results, thus potentially overlooking most of the possible true associations. In the future, longitudinal studies with larger sample sizes could be conducted to track and determine causal relationships between exposures and outcomes and to gain insight into the potential mechanisms of action of multiple metal exposures affecting inflammatory states in vivo.

## Conclusion

In conclusion, plasma metals Cu and Mn were positively correlated with immunoinflammatory markers SII, NLR, and PLR. While plasma metal Fe was negatively correlated with immunoinflammatory markers SII, NLR, and PLR.

### Supplementary Information

**Supplementary Material 1. **

## Data Availability

Data used in the current study are available from the corresponding author upon reasonable request.
