# Correction: Association between multiple-heavy-metal exposures and systemic immune inflammation in a middle-aged and elderly Chinese general population

**DOI:** 10.1186/s12889-024-19124-2

**Published:** 2024-06-30

**Authors:** Linhai Zhao, Yanfei Wei, Qiumei Liu, Jiansheng Cai, Xiaoting Mo, Xu Tang, Xuexiu Wang, Lidong Qin, Yujian Liang, Jiejing Cao, Chuwu Huang, Yufu Lu, Tiantian Zhang, Lei Luo, Jiahui Rong, Songju Wu, Wenjia Jin, Qinyi Guan, Kaisheng Teng, You Li, Jian Qin, Zhiyong Zhang

**Affiliations:** 1 Department of Occupational and Environmental Health, School of Public Health, Guangxi Medical University, Nanning, Guangxi Zhuang Autonomous Region China; 2 School of Public Health, Guilin Medical University, Guilin, Guangxi Zhuang Autonomous Region China; 3 Department of Epidemiology, School of Public Health (Guangdong Provincial Key Laboratory of Tropical Disease Research), Southern Medical University, Guangzhou, Guangdong China; 4 Guangxi Key Laboratory of Entire Lifecycle Health and Care, Guilin Medical University, Guilin, Guangxi Zhuang Autonomous Region China; 5 Guangxi Colleges and Universities Key Laboratory of Prevention and Control of Highly Prevalent Diseases, Guangxi Medical University, Nanning, Guangxi Zhuang Autonomous Region China; 6 Guangxi Key Laboratory of Environment and Health Research, Guangxi Medical University, Nanning, Guangxi Zhuang Autonomous Region China; 7 Key Laboratory of Longevity and Aging‑related Diseases of Chinese Ministry of Education, Guangxi Medical University, Nanning, Guangxi Zhuang Autonomous Region China

**Correction:** **BMC Public Health 24, 1192 (2024)**

**https://doi.org/10.1186/s12889-024-18638-z**

In the original publication of this article [1] there was an error in Figure 3. The rightmost values of the P trend and the corresponding asterisk symbol were not visible. The incorrect and correct figure are shown in this correction article. The original article has been updated.

**Incorrect figure 3**


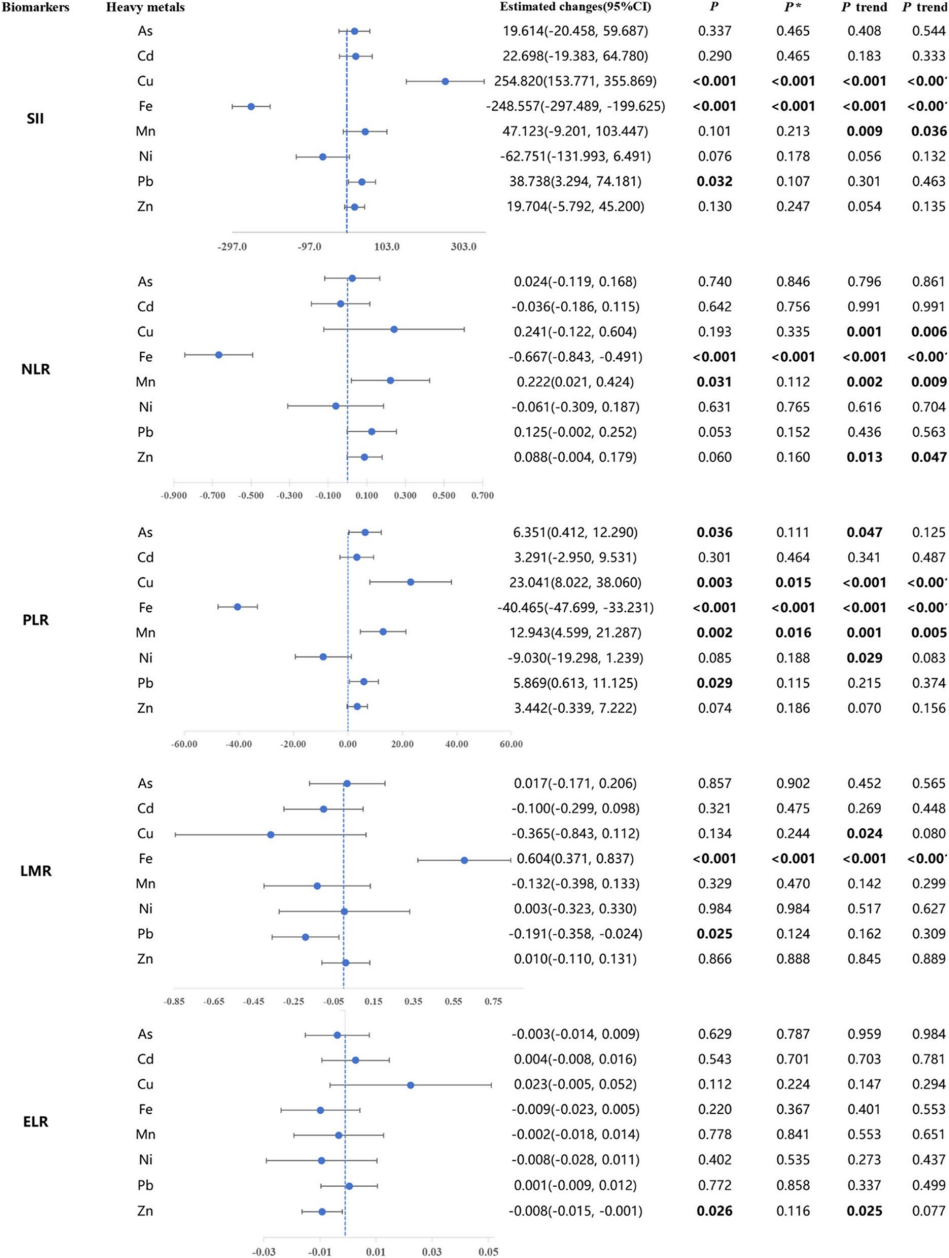


**Correct figure** 3

**Fig. 3:**
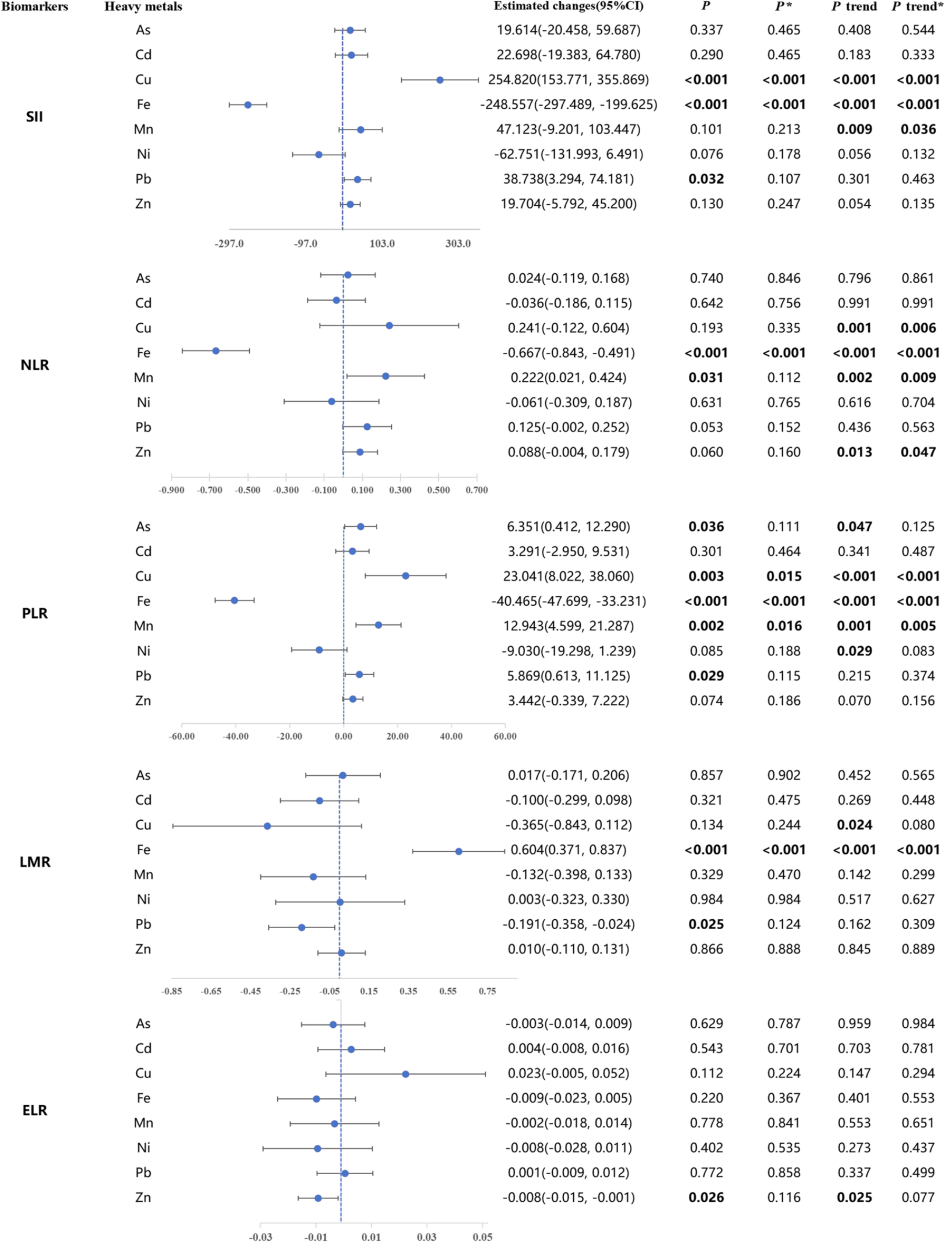
Results of Generalized Linear Regression Analysis of the Association Between Single Plasma Heavy Metals (log_10_ Converted Values) and immune inflammation biomarkers. All above are adjusted for gender, age, Ethnicity, Educated, BMI, Smoking, Alcohol consumption, Marital status, Diabates, Dyslipidemia, and Hypertension. *β* is the change in the standardized systemic inflammatory index per 1 SD increase in log_10_-converted plasma heavy metal concentrations. The value of *β* at the blue dashed line is 0. *P* trend was examined by using the median of each quartile of plasma heavy metals as a continuous variable in the model. ^*^P value/*P* trend in multiple testing
