# Supplementary material for: Association between multiple-heavy-metal exposures and systemic immune inflammation in a middle-aged and elderly Chinese general population
Source: BMC Public Health. 2024 Apr 29;24:1192. doi: 10.1186/s12889-024-18638-z (PMC11057124; doi:10.1186/s12889-024-18638-z)
Supplement: Supplementary file 1 — Supplementary Material 1. [file 12889_2024_18638_MOESM1_ESM.docx]

**Questionnaire on basic demographic information**

**serial number:**

1.What is your name?

2. Where is your birthplace? (Specific to the village)

3. Where is your current residence? (Specific to the village)

4. Length of time you have lived in your current residence:

□ Within 1 year □ 1 to 3 years (not included) □ 3 to 5 years (not included) □ 5 to 10 years (not included) □ More than 10 years

5. Home address:

6. Gender:

□Male □Female

7. How old are you? years old

8. What is your date of birth?

9. May I ask what ethnicity you are?

□Han □Zhuang □Yao □Mulao □Dong □Others:

10. What is your current marital status?:

□Unmarried □Married □Widowed □Divorced □Cohabiting □Separated □Other:

11. What is your level of education?

□Not receiving formal education □Not graduated from elementary school □Graduated from elementary school □Junior high school □High school / technical secondary school □College and above

12. Do you smoke? □No □Yes (Please go to question 12.1)

12.1 Type of tobacco and amount smoked: □Mechanized cigarettes: cigarettes/day □Hand-rolled cigarettes: cigarettes/day □Dry cigarettes/pipe: two/day □Other:

12.2 Years of smoking: Years

13. Do you drink alcohol? □No □Yes (please go to question 13.1)

13.1 Type of alcohol consumption: □liquor (≥42 degrees) □liquor (<42 degrees) □home-brewed rice wine □yellow wine □beer □wine □other:

13.2 Amount of alcohol consumed: □□. □□ two/day

13.3 Drinking frequency:□day/week

13.4 Years of drinking alcohol: Years

14. Have you ever been hospitalized or operated for any disease: □ No □ Yes (please go to question 14.1)

14.1 What kind of illnesses have you ever suffered from:

①：Name of disease： ; Date of illness: years month days

②：Name of disease： ; Date of illness: years month days

③：Name of disease： ; Date of illness: years month days

④：Others： ; Date of illness: years month days

15. Have you taken any medications in the last 2 weeks? □ No □ Yes (please go to question 15.1)

15.1 What kind of medications have been taken:

□ lipid-lowering drugs □ antihypertensive drugs □ oral hypoglycemic drugs □ insulin □ painkillers □ anticoagulants □ sleeping pills □ medications for asthma □ diuretics □ antibiotics □ hormonal drugs □ thrombolytics □ aspirins □ others:

16. Do you have a chronic disease? (multiple choice):

□ No □ High blood pressure □ Diabetes mellitus □ Cerebrovascular disease □ Chronic obstructive pulmonary disease □ Rheumatism □ Osteoarthropathy □ Osteoporosis □ Cancer □ Others:
